# Barriers to medication adherence by caregivers of children with leukemia: an observational study

**DOI:** 10.1590/1984-0462/2024/42/2022214

**Published:** 2023-10-23

**Authors:** Fernanda Alves França, Ana Catarina Fernandes Figueredo, Luiza Tessmann, Valdenize Tiziani, José Carlos Martins Córdoba, Isis Magalhães, Noêmia Urruth Leão Tavares, Patrícia Medeiros-Souza

**Affiliations:** a Universidade de Brasília, Brasília, DF, Brazil.; b Hospital da Criança de Brasília José Alencar, Brasília, DF, Brazil.; c Secretaria de Estado de Saúde do Distrito Federal, Brasília, DF, Brazil.

**Keywords:** Child, Caregivers, Leukemia, Treatment adherence, Criança, Cuidadores, Leucemia, Adesão ao tratamento

## Abstract

**Objective:**

To identify barriers to adherence to home oral maintenance chemotherapy in children with leukemia treated at a specialized cancer center.

**Methods:**

We used the Brief Medication Questionnaire (BMQ) as a tool for screening barriers to adherence. The level of adherence was calculated considering at least one positive response in each BMQ domain, defined as Regimen Screen, Belief Screen, and Recall Screen. A positive screening for belief barriers (PSB) indicates that the caregiver reports not understanding the medication's mechanism of action and adverse effects.

**Results:**

Three important barriers to adherence were identified: beliefs, number of children of the caregiver, and age of the caregiver. The primary caregivers included 32 mothers (80%), four fathers (10%), three grandmothers (7.5%), and one unrelated caregiver (2.5 %). Most caregivers with a PSB were mothers. A PSB indicates that the caregiver reports not understanding the medication's mechanism of action and adverse effects. Caregivers with two or more children (median, three) had more barriers to adherence. Caregivers with potential non-adherence tended to be older than those with potential adherence, although without statistical significance (p=0.079, Mann-Whitney U test).

**Conclusions:**

The main barriers to adherence to home oral maintenance chemotherapy in children with leukemia identified through interviews with their caregivers, most often mothers, were lack of understanding of the treatment regimen, a greater number of children, and older age.

## RESUMO

**Objetivo:**

Identificar barreiras de adesão ao tratamento de manutenção da quimioterapia via oral domiciliar, em uma amostra de crianças diagnosticadas com leucemia atendidas em um serviço especializado em oncologia.

**Métodos:**

O *Brief Medication Questionnaire* (BMQ) foi utilizado como instrumento de coleta para a identificação de barreiras de adesão. O nível de adesão foi calculado considerando-se pelo menos uma resposta positiva no domínio do BMQ, definido como regime, crença e recordação. Uma crença positiva mostra que o cuidador reporta não entender o mecanismo de ação e os efeitos adversos.

**Resultados:**

Três importantes barreiras de adesão foram identificadas, incluindo crença, o número de filhos do casal e a idade dos cuidadores. A mãe como principal responsável pelo tratamento da criança apresentou frequência maior entre as pessoas com rastreamento positivo para barreiras de crenças (BPC). Crença positiva significa que o cuidador relata não entender o mecanismo de ação dos medicamentos e os efeitos adversos. Quanto ao número de filhos, o estudo mostrou que quanto mais filhos (dois filhos ou mais, mediana=três) maior a barreira de adesão. Houve tendência de responsáveis com potencial não adesão serem mais velhos que os responsáveis com potencial adesão, embora sem significância estatística ao nível de significância de 5% (p=0,079, teste U de Mann-Whitney).

**Conclusões:**

As principais barreiras de adesão dos cuidadores de crianças com leucemia ao tratamento medicamentoso de manutenção foram dificuldades relatadas pelos cuidadores, na maioria das vezes as mães, que não entenderam como o medicamento funcionava, o número de filhos — quanto mais filhos menor a adesão — e a idade dos cuidadores. Cuidadores mais velhos aderiram menos ao tratamento prescrito.

## INTRODUCTION

Childhood cancer is the leading cause of disease-related death, approximately 8%, in children and adolescents in Brazil. Recent data show that leukemia is the most commonly diagnosed malignancy affecting children and adolescents.^1^ Access to diagnosis of children with leukemia has increased significantly in the last 40 years, and cure rates of children treated in specialized centers reach 80% in high-income countries.^1^

Leukemia is defined as a primary malignancy of hematopoietic stem cells. Because these cells are genetically altered in leukemia, they lose their differentiation ability while maintaining their proliferative capacity, with subsequent reduction in the production of healthy hematopoietic elements.^2^ Leukemia can be divided into subgroups based on the morphology of the affected cells and speed of disease progression, being classified as acute or chronic.^3^ They can be further divided into subgroups according to the type of affected cells, being characterized as lymphocytic leukemia, myeloid leukemia, and nonspecific or combined types.^3^

Conventional leukemia treatment is divided into phases. The first phase aims to eliminate the pool of abnormal cells and is called remission induction. Once cytological remission has been achieved, less than 5% of bone marrow cells are expected to be leukemic with restoration of normal hematopoiesis.^4^ However, despite no visible evidence of disease on cytology, studies have reported residual leukemia cells in the body, which requires further treatment to prevent relapse.^4^ The last phase is called maintenance, characterized by continuous treatment for several months.^4^

Multiple factors should be considered during treatment. Examples include the complexity of dosing regimens, the duration and cost of treatment, adverse events, subgroups with poor prognosis, a low health literacy population, cognitive and functional impairment, beliefs, concerns, patients’ and caregivers’ perceptions of health and treatment status, and lack of family and social support, among others.^5^ These factors may hinder treatment adherence, compromising clinical outcomes and potentially leading to relapse that might increase caregiving demands.^5^

Barriers to adherence can be considered a public health problem because of consequences such as worsening of the disease, increased adverse events, and drug treatment failure, which may lead to disease progression and the need for hospitalization.^6^

The primary objective of this study was to identify barriers to adherence to home oral maintenance chemotherapy in children with leukemia treated at a specialized cancer center.

## METHOD

The present study outlined the epidemiological profile of the main adherence barriers in children with leukemia treated at a referral hospital in Brasília, Brazil. We conducted a cross-sectional, observational, analytical study of 40 children diagnosed with leukemia between one and ten years of age who reached the maintenance phase of the chemotherapy protocol and were not hospitalized at the time of the study by convenience sample.

Data were collected through an interview with the person responsible for the child's treatment (caregiver) using the adapted version of the Brief Medication Questionnaire (BMQ)^7^ translated into Portuguese and validated in Brazil as a tool for screening barriers to adherence.

A total of 49 children diagnosed with leukemia receiving maintenance chemotherapy were considered eligible for the study. Eight children were excluded from the study because they were older than ten years, and one child was excluded because the caregiver refused to complete the questionnaire. Therefore, the final sample consisted of 40 children.

Descriptive and association analyses were performed for levels of adherence and for levels of adherence by BMQ domain. The level of adherence was calculated considering at least one positive response in each BMQ domain, defined as Regimen Screen, Belief Screen, and Recall Screen. Responses to the questionnaire were categorized into levels of adherence according to the number of positive responses in any of the BMQ domains: high adherence (none), probable high adherence (1), probable low adherence (2), and low adherence (3 or more). A positive screening for belief barriers (PSB) indicates that the caregiver reports not understanding the medication's mechanism of action and adverse effects.^7^

Qualitative variables characterizing the children and caregivers were expressed as frequency (n) and percentage (%) and associated using Pearson's chi-square test with continuity correction or Monte Carlo simulation if necessary (at least one cell was expected to have a value < 5). Quantitative variables were associated using the nonparametric Mann-Whitney U test and Kruskal-Wallis test for independent samples, considering these variables did not present normal distribution by the Kolmogorov-Smirnov test. Data were analyzed using IBM Statistical Package for the Social Sciences — SPSS, version 23, 2015. The significance level was set at 5% for all analyses.

The study followed the standards for research involving human subjects, set forth in Resolution 466/12 of the Brazilian National Health Council, and was approved by the Research Ethics Committees of the University of Brasília School of Health Sciences (approval number 2.808.180; August 11, 2018) and Health Science Teaching and Research Foundation (Certificate of Presentation for Ethical Appreciation [CAAE] number 87652818.5.3001.5553 and approval number 2.979.001; October 24, 2018). All caregivers provided written informed consent to be interviewed for the study.

## RESULTS

The sociodemographic characteristics of the children and caregivers participating in the study are shown in Table 1. The primary caregivers included 32 mothers (80%), four fathers (10%), three grandmothers (7.5%), and one unrelated caregiver (2.5%). Mothers as caregivers reported a more PSB in treatment (n=36.192, 90.5%) than did grandmothers (n=1.904, 4.8%).

**Table 1:** Descriptive analysis of sociodemographic variables of caregivers and children diagnosed with leukemia between 1 and 10 years of age in the maintenance phase of the chemotherapy protocol treated at Hospital da Criança de Brasília José Alencar, Brazil.

Variable		n	%
Sex			
	Male	24	60.0
Pathology			
	ALL	38	95.0
	AML	2	5.0
Race			
	White	16	40.0
	Mixed-race	16	40.0
	Black	4	10.0
	Asian descent	4	10.0
Private health insurance			
	No	34	85.0
Sex of the caregiver			
	Female	35	87.5
Marital status			
	Single	8	20.0
	Cohabiting	12	30.0
	Married	15	37.5
	Divorced	5	12.5
Level of education			
	Incomplete elementary school	10	25.0
	Complete elementary school	2	5.0
	Incomplete high school	8	20.0
	Complete high school	13	32.5
	College, no degree	2	5.0
	Bachelor's degree	3	7.5
	Master's degree or higher	2	5.0
Currently working			
	No	28	70.0
Monthly income*			
	From 1 to < 2 mms (R$ 937.00 to R$ 1,873.99)	24	60.0
	From 2 to < 4 mms (R$ 1,874.00 to R$ 3,747.99)	10	25.0
	From 4 to < 6 mms (R$ 3,748.00 to R$ 5621.99)	3	7.5
	From 6 to < 10 mms (R$ 5,622.00 to R$ 9,369.99)	2	5.0
	≥ 10 mms (R$ 9,370.00 or more)	1	2.5
Race of the caregiver			
	White	9	22.5
	Mixed-race	20	50.0
	Black	6	15.0
	Asian descent	5	12.5
Degree of kinship			
	No kinship	1	2.5
	Mother	32	80.0
	Father	4	10.0
	Grandmother	3	7.5
Place of residence			
	Federal District	28	70.0
	State of Goiás	10	25.0
	State of Minas Gerais	1	2.5
	State of Rondônia	1	2.5
	Total	40	100.0

ALL: acute lymphoblastic leukemia; AML: acute myeloid leukemia.

* Monthly income was reported as Brazilian minimum monthly salaries; the Brazilian minimum monthly salary (mms) denotes government regulation for a minimum monthly rate paid for a worker who works, on average, 44 hours a week for 4 weeks in a month.

The responses to the BMQ questionnaire were associated with qualitative variables (Table 1) and quantitative variables (Table 2) to evaluate factors potentially associated with lower adherence to home oral maintenance chemotherapy among children.

**Table 2:** Association analysis between quantitative sociodemographic variables and adherence to home oral maintenance chemotherapy of children diagnosed with leukemia between 1 and 10 years of age in the maintenance phase of the chemotherapy protocol treated at Hospital da Criança de Brasília José Alencar, Brazil.

Adherence							
	Probable high adherence		Probable low adherence		Low adherence		p-value*
	Median	IQR	Median	IQR	Median	IQR	
Age	4.5	4.0	5.0	3.5	5.0	3.0	0.827
Age of the caregiver	30.0	8.3	30.0	16.8	37.5	15.3	0.524
Number of children	1.0	0.3	2.0	2.0	2.0	1.8	0.031

IQR: interquartile range;

* Nonparametric Kruskal-Wallis test.

**Figure 1:**
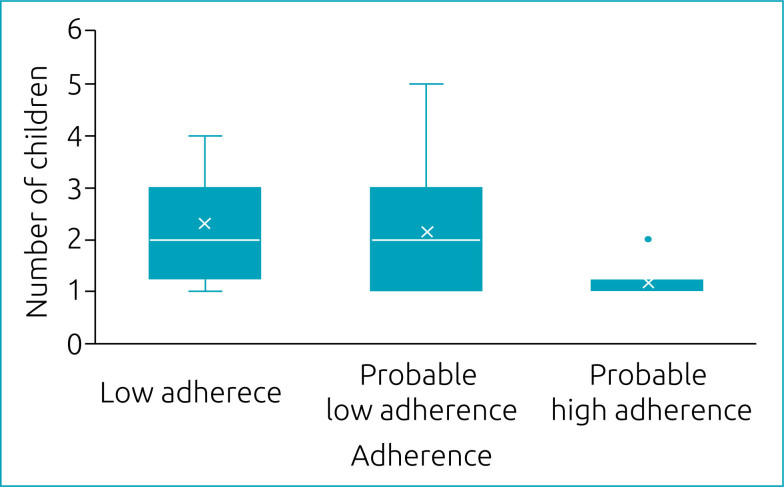
Association between the number of children of the caregiver and adherence to home oral maintenance chemotherapy of children diagnosed with leukemia between 1 and 10 years of age treated at Hospital da Criança de Brasília José Alencar, Brazil.

Table 2 shows that only the variable ‘number of children’ was significantly associated with adherence to treatment by the caregiver. Caregivers with probable low adherence and low adherence had significantly more children than those with probable high adherence (Table 2 and Figure 1).

**Figure 2:**
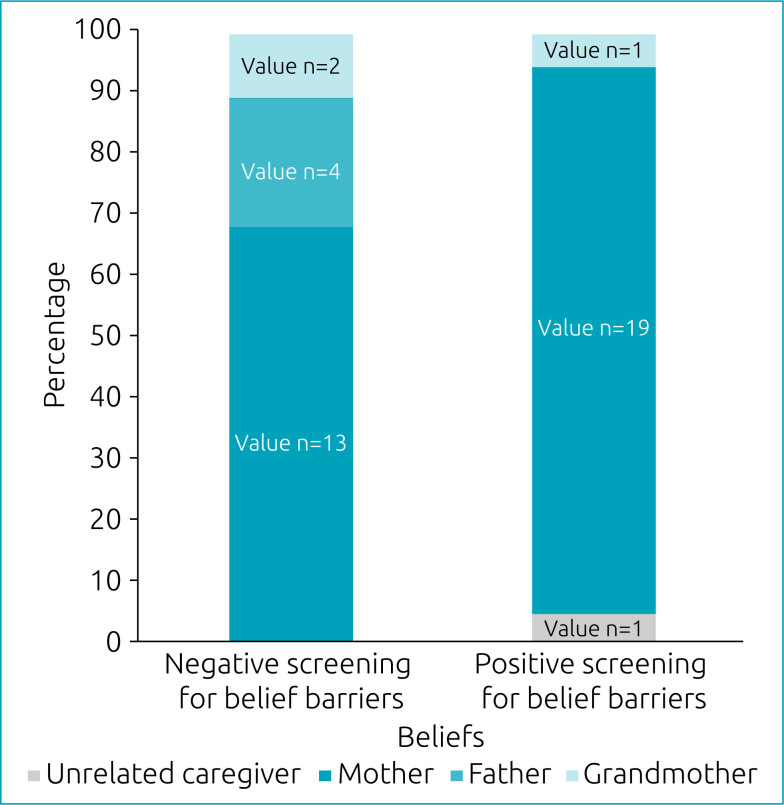
Association between the degree of kinship of the caregiver and Belief Screen of adherence to home oral maintenance chemotherapy of children diagnosed with leukemia between 1 and 10 years of age treated at Hospital da Criança de Brasília José Alencar, Brazil.

No qualitative sociodemographic variable of the caregivers was significantly associated with the Belief Screen in children with leukemia receiving home oral maintenance chemotherapy. The degree of kinship of the caregiver showed a strong tendency toward association with the Belief Screen, although without statistical significance (p=0.056, Pearson's chi-square test). Most caregivers with a PSB were mothers (Figure 2).

**Figure 3:**
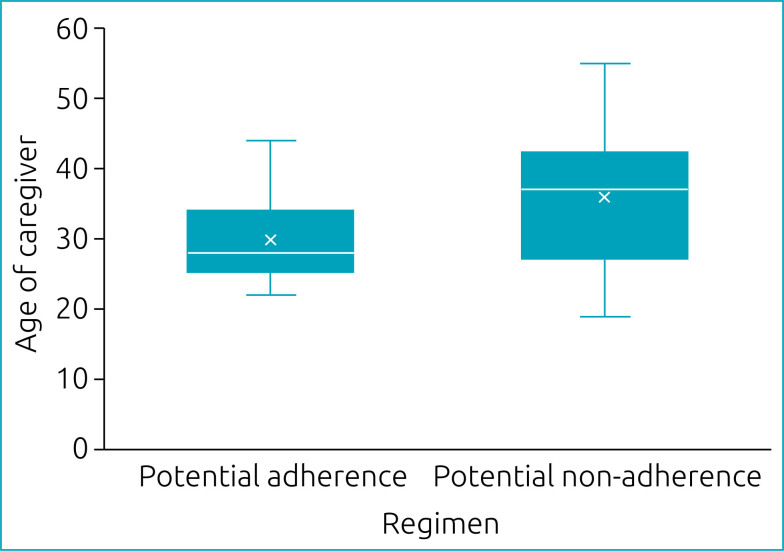
Association between age of the caregiver and Regimen Screen of adherence to home oral maintenance chemotherapy of children diagnosed with leukemia between 1 and 10 years of age, treated at Hospital da Criança de Brasília José Alencar, Brazil.

Figure 3 shows that caregivers with potential non-adherence tended to be older than those with potential adherence, although without statistical significance (p=0.079, Mann-Whitney U test).

## DISCUSSION

Adherence barriers identified in the study were associated with beliefs, number of children, and drug regimen in relation to the age of the caregiver. In most cases, the primary caregiver was the mother (n=32, 80%). Mothers also reported a more PSB than did other caregivers. The results indicate the main areas that should be addressed by the multidisciplinary team to improve medication adherence.^7^

Regarding the PSB, Bastos^8^ conducted a qualitative study based on discourse analysis seeking to identify factors associated with anticipatory grief when caregivers, who most often are mothers, are told that their child has cancer or requires palliative care. The results showed that, while the mother is taking care of her child, she suffers from the belief that her child may die during treatment, leading to distress that may be related to the belief that the treatment is not working.^8^

A systematic review of health literacy, conducted at the University of Amsterdam in 2015, showed that people with low health literacy are less likely to understand health information.^9^ Our findings are consistent with these results, since there was a PSB regarding the understanding of the treatment regimen among the caregivers who had not completed elementary school (n=10, 25%).

Treating childhood cancer is complex. According to a quantitative analytical study conducted in 2019 in the oncology unit of a public hospital in Pernambuco, Brazil, patients with acute lymphoblastic leukemia are treated with several drugs, suggesting polypharmacy as the main factor leading to potential drug-drug interactions.^10^ This may be related to the PSB of mothers, which indicates they do not actually understand the mechanism of action of the simultaneous use of multiple drugs in the treatment regimen.

A qualitative study of ten women conducted in two phases from 2015 to 2018 using structured interviews, in addition to identifying that the mother was the primary caregiver of children with congenital syndrome (microcephaly associated with Zika virus infection),^11^ also showed that mothers were overwhelmed by caring for their sick children, facing problems related to stress and responsibilities inherent in the treatment.^11,12^ Generally, mothers withdraw from their other children, leave their job and social life to dedicate themselves fully to the sick child,^13^ and, as a consequence, they often experience mental health problems, high levels of stress, anxiety, and depression, decreased sense of well-being, and decreased quality of life.^14^

The number of children of the caregiver had a negative impact on adherence to treatment. Our study showed that caregivers with two or more children (median, three) had more barriers to adherence. A possible explanation for this association is that attention was not focused on the sick child, being shared between the sick sibling and healthy siblings.^15^ Another important issue to be addressed is that having a child with cancer changes the family dynamics, as there is a considerable impact on the spousal relationship and on the care of healthy siblings.^16^ The primary caregiver, usually the mother, feels guilty about the series of events that happen to the sick child, in addition to feeling that she is not fulfilling her responsibilities as a mother toward her other healthy children.^16^

A childhood cancer diagnosis can be considered a negative life event that interacts with aspects of personal life, environment, and family system, influencing the daily life of the entire family.^17^ The family daily routine often changes after a cancer diagnosis, which directly affects healthy siblings, who feel they are a burden on parents, and becomes an additional concern for parents, who try to share the attention between all children.^16^ However, due to the severity of the disease, parental attention tends to be focused on the child with cancer, which may cause discomfort and psychological problems in the healthy siblings who also need care.^17^ Once again, the mother assumes responsibility and feels guilty for not being able to be present in the lives of all her children.^15–17^

The present study also identified that, in the Regimen Screen, younger caregivers showed higher adherence to the prescribed leukemia treatment (age < 30 years; median, 29 years). It may be suggested that older people have habits, thoughts, and beliefs of their own that make it difficult to follow the physician's and multidisciplinary team's instructions about pill splitting, disposal, and administration as well as drug-drug and drug-food interactions, which are correctly performed by health professionals. Therefore, caregivers’ difficulty following instructions becomes a barrier to adherence that worsens with advancing age. However, a study of six caregivers of older people conducted in Rio Grande do Sul, Brazil, showed that older caregivers were more resilient, that is, better able to adjust to change, which may facilitate adherence to treatment.^18^

This study has limitations. The method used considers polypharmacy a barrier to adherence. However, cancer treatment has multiple therapeutic targets, making monotherapy difficult. Another limitation is the questionnaire used, as the BMQ is not a standardized instrument to screen pediatric patients undergoing cancer treatment. The small number of caregivers interviewed can also be considered a limitation, in addition to the fact that the study was conducted in a single center. Therefore, further studies with a larger sample size are needed. Nevertheless, only a few patients were excluded from the study, and we were able to outline a profile of the pediatric population with leukemia treated at our institution and identify treatment-related factors that need to be addressed. Considering that the main barrier to adherence identified in this study was the understanding of the treatment regimen, additional studies are suggested to assess the level of patient satisfaction^19^ in this population.

In this context, a descriptive cross-sectional study was conducted in a reference outpatient clinic in Brasília between 2017 and 2019 to evaluate the level of satisfaction of adolescents’ caregivers with information received on the use of psychotropic drugs using the Satisfaction with Information about Medicines Scale (SIMS), and the results showed that most caregivers were dissatisfied with the information about treatment.^20^ Although we analyzed a different population in the present study, we ended up identifying the same problem of lack of caregiver understanding, especially of mothers (80%), as a barrier to adherence to treatment, thus highlighting the urgent need of additional studies to enhance caregiver understanding of leukemia treatment so that they can act as agents in the treatment of these children.^21^

Another important factor to be evaluated in further studies is the follow-up that supports the use of event-free survival and overall survival in pediatric oncology after maintenance treatment of acute lymphocytic leukemia.

The main barriers to adherence to home oral maintenance chemotherapy in children with leukemia identified through interviews with their caregivers, most often mothers, were lack of understanding of the treatment regimen, a greater number of children, and older age.

## Data Availability

The database that originated the article is available with a corresponding author.
